# A Tale from TGF-β Superfamily for Thymus Ontogeny and Function

**DOI:** 10.3389/fimmu.2015.00442

**Published:** 2015-09-10

**Authors:** Arnon Dias Jurberg, Larissa Vasconcelos-Fontes, Vinícius Cotta-de-Almeida

**Affiliations:** ^1^Laboratory on Thymus Research, Oswaldo Cruz Institute, Oswaldo Cruz Foundation (Fiocruz), Rio de Janeiro, Brazil; ^2^Graduate Program in Cell and Developmental Biology, Institute of Biomedical Sciences, Federal University of Rio de Janeiro, Rio de Janeiro, Brazil

**Keywords:** TGF-β, BMP, thymus, thymopoiesis, T cell development

## Abstract

Multiple signaling pathways control every aspect of cell behavior, organ formation, and tissue homeostasis throughout the lifespan of any individual. This review takes an ontogenetic view focused on the large superfamily of TGF-β/bone morphogenetic protein ligands to address thymus morphogenesis and function in T cell differentiation. Recent findings on a role of GDF11 for reversing aging-related phenotypes are also discussed.

## Introduction

The adaptive immune system evolved as a complex set of defense mechanisms amplified by the specificity properties of antigen receptor-bearing B and T lymphocytes (1). Following blood trafficking into the thymus, bone marrow-derived lymphoid progenitors become committed to T cell lineage development. Within this organ, cell specialization occurs gradually in a manner that T cell development results in the generation of conventional CD4 and CD8 αβ T cells along with natural killer T cell (NKT; an innate-like T cell subpopulation), regulatory T cell (Treg), and γδ T cell subsets (2). Classically, commitment to T cell lineage was found to rely on the Delta-class Notch ligand Delta-like 4 (DLL4) and the interleukin-7 (IL-7) along with kit and flt3 ligands at stages usually prior to TCRβ chain assembling (3–6). Branching into distinct paths can be observed throughout the mainstream developmental pathway, from the double-negative (DN; CD4^−^CD8^−^) T cell precursors to the highly expanded double-positive (DP; CD4^+^CD8^+^) cells, and the resulting mature single-positive (SP; CD4^+^CD8^−^ or CD4^−^CD8^+^) stages. Thus, at specific niches, the thymus provides to developing T cells signals that trigger a series of ordered events leading to cell proliferation, TCR gene rearrangements, and selective checkpoints along with massive cell death (7). Altogether, these events culminate in a proper repertoire of distinct and specialized mature thymocyte subpopulations able to emigrate to the periphery. In this review paper, we highlight the role of members of the large transforming growth factor-β (TGF-β) superfamily (Box 1) during thymic ontogeny, thymic epithelial cell (TEC) differentiation and function, as well as T cell maturation. Lastly, we discuss recent information on a possible regenerative potential of TGF-β ligands to rescue aging-related thymus atrophy.

Box 1 **Multiple roads for signaling by TGF-β superfamily members.**

The TGF-β superfamily comprises TGF-β1–3, bone morphogenetic proteins (BMPs), growth and differentiation factors (GDFs), Nodal, activins/inhibins, Müllerian inhibiting substance (MIS)/anti-Müllerian hormone (AMH), and Lefty. These ligands were initially grouped accordingly to the functional roles observed following their original identification (8–11). As it became clear that most ligands play multiple functions depending on cell type, developmental stage, or tissue conditions, they are now classified by sequence similarity and the downstream pathway they activate (12). Each family member has an overall basic structure, in which inactive forms are produced with an N-terminal secretion peptide and a large propeptide domain known as latency-associated peptide (LAP). Cleavage of the propeptide domain by proprotein convertases releases a mature domain at the C-terminus, which eventually dimerizes (13). The propeptide domain has major regulatory roles. It influences protein stability and functions as chaperone during secretion, also mediating diffusion through interactions with the extracellular matrix and inhibiting the active peptide form even after cleavage (14–16).

**Figure 1:**
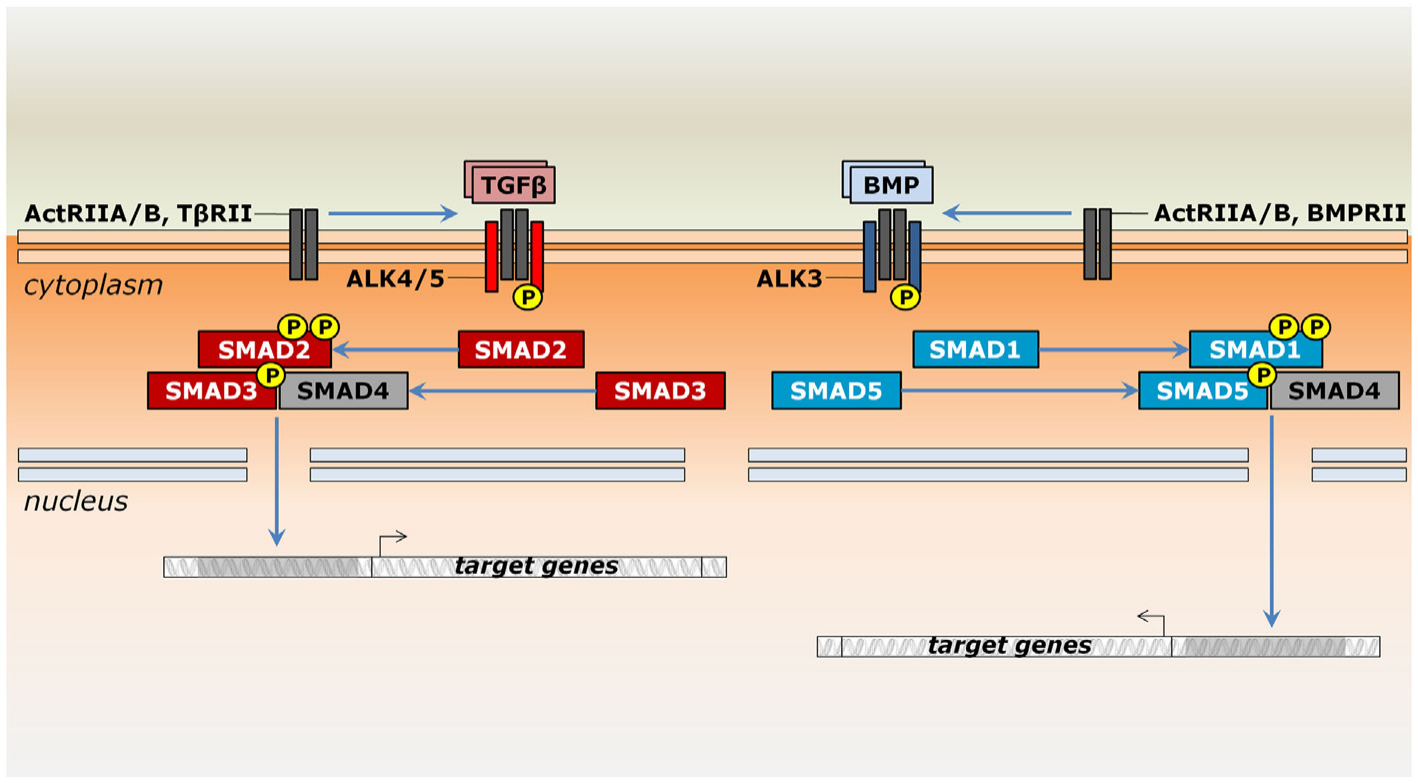
**Signaling by ligands of the TGF-β superfamily in the thymus**. Members of the TGF-β superfamily may signal by either the TGF-β (reddish) or the BMP branch (bluish). Upon binding to type II serine/threonine receptors occurs the recruitment of type I receptors, which further phosphorylate Smad proteins. Whereas ActRIIA and ActRIIB may be shared between both pathways, TβRII and BMPRII are specific to TGF-β and BMP signaling, respectively. In general, Smad2 and Smad3 relay signals from the Alk4, Alk5, and Alk7 receptors, while Smad1, Smad5, and Smad8/9 are phosphorylated by Alk2, Alk3, and Alk6 receptors. However, Alk2, Alk6, and Alk7 are not expressed during thymocyte maturation. Modulation of gene expression occurs after Smad complex translocates into the nucleus and depends on the interaction with additional protein complexes (not shown).

Signaling by TGF-β superfamily members occurs through a similar mechanism, but operates with distinct components. Ligands bind single-pass transmembrane receptor serine/threonine kinases, which relay the signal for intracellular effectors capable of translocating into the nucleus to modulate gene transcription (Figure 1). More specifically, these receptors are classified into two structurally similar types. Ligand binding occurs only through type II receptors, which then recruit and phosphorylate type I receptors [e.g., Ref. (17, 18)]. Type II receptors, such as ActRII (*Acvr2a*) or ActRIIB (*Acvr2b*), may take part in many distinct pathways or may be specific for a given group of ligands, such as AMHR2 (*Amhr2*) for MIS/AMH, BMPRII (*Bmpr2*) for most BMPs and Gdf9, and TβRII (*Tgfbr2*) for TGF-βs (19, 20). Type I receptors are also known as activin receptor-like kinases (ALKs) due to their sequence similarity to activin receptors (21). These receptors are usually specific to a more restricted set of ligands. For instance, Nodal, Gdf1, Gdf11, activins, and inhibins bind ActRII to recruit Alk4 (*Acvr1b*) and Alk7 (*Acvr1c*) or they bind ActRIIB to recruit either Alk4, Alk7, or Alk5 (*Tgfbr1*) (19). Together, type II and type I receptors form a heterotetrameric complex, in which the type I receptor further phosphorylates intracellular effectors of the Smad family (22). Depending on the ligand/receptor complex they are responding to, receptor-activated Smads (R-Smads) can be subdivided into two groups: a BMP-related set gathers Smad1, Smad5, and Smad9 (formerly Smad8), whereas Smad2 and Smad3 are responsive to TGF-β-related signals (Figure 1). An N-terminal MH1 domain negatively regulates the MH2 domain, being indispensable for Smad translocation into the nucleus and DNA binding (23–25). However, these functional properties do not hold true for all R-Smads. In particular, Smad2 seems to interact to DNA only indirectly (24).

A common mediator Smad (co-Smad), or Smad4, integrates signals from both branches by associating with the R-Smads (Figure 1). They form transcriptional complexes able to translocate into the nucleus (26–28). Nuclear transportation of Smads depends on accessory proteins, particularly importins, exportins, and nucleoporins (29, 30). The presence of DNA molecules harboring Smad-binding elements favors heterodimerization between R-Smads and co-Smad (28). They ultimately associate with cell-type-specific transcription factors and co-activators to regulate a plethora of target genes (31).

Regulation of Smad activity occurs through multiple mechanisms (32). Two inhibitory Smads (I-Smads) impair signaling by competing with R-Smads for receptors or by co-Smad interaction (33). For instance, Smad6 forms stable interactions with type I receptors, blocking phosphorylation of Smad2 and Smad1, but not Smad3 (34, 35). Similarly, Smad7, the other I-Smad member, also binds type I receptors and suppresses further phosphorylation by targeting them for proteasome-dependent degradation (35, 36). The available literature on the molecular interactions of TGF-β superfamily members is vast, but not in the scope of this review. Further information can be found elsewhere (33, 37–40).

## TGF-β Signaling and Thymus Formation

**Figure 2:**
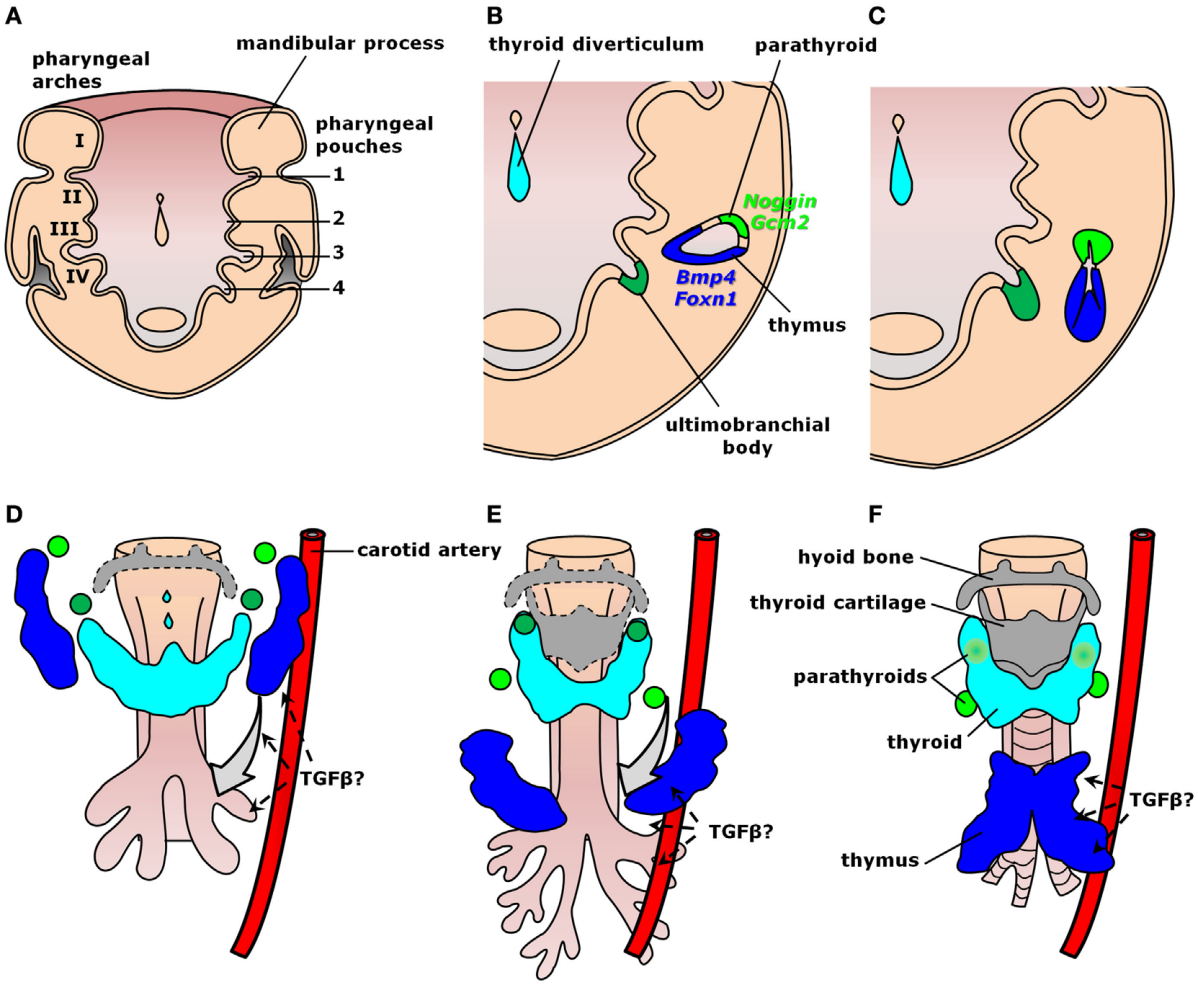
**Signaling by TGF-β superfamily members during thymus organogenesis**. Schematic representation of thymus formation at different stages of development. **(A–C)** Thymus specification viewed dorsally at the ventral half of the pharyngeal region. **(A)** The common parathyroid–thymus primordium arises from the third pharyngeal pouch endoderm. **(B)** Within each anlage, mTEC precursors line a central lumen surrounded by a dorso-rostralmost domain expressing the BMP-antagonist *Noggin* and the parathyroid specific gene *Gcm2* (light green), whereas the ventro-caudalmost domain expresses *Bmp4* and the thymus-specific gene *Foxn1* (blue). **(C)** Each primordium grows in size while proliferating cells fill the rudiment lumen, later colonized by lymphocyte precursors to produce an inner medulla. **(D–F)** Thymus migration toward the heart. The inferior parathyroid (light green) and the thymus (blue) primordia are gradually resolved as they migrate downwards. **(D)** TGF-β cues from the endothelium of pharyngeal blood vessels (e.g., carotid arteries) seem to orient thymic and parathyroid migration toward their final location. **(E)** The third pharyngeal pouch-derived thymic and the inferior parathyroid rudiments pass by the primordia of the superior parathyroid (dark green), which migrate only a short distance downward the tracheal region. **(F)** Fusion of the thymic primordia occurs at the midline just above the developing heart (not shown) [modified from Ref. (45)].

Organogenesis relies on well-organized interactions between distinct germ layers and differentiating cell types controlled by intricate molecular hierarchies. Thymus development occurs from common parathyroid bilateral rudiments in the epithelial endodermal lining of the third pharyngeal pouch around embryonic days (E) 9.0–9.5 in mice and early week 5 in humans (Figure 2A) (41–44). As growth continues through E10.5 in mice and early week 6 in humans, the contact between the third pharyngeal pouch and the third pharyngeal cleft ectoderm determines paired organ primordia with stratified epithelium and a central lumen lined by precursors of medullary thymic epithelial cells (mTECs). These cells are characterized by the expression of both claudin-3/4 and cytokeratin-5 (K5) (46, 47). Further development of thymic medulla also depends on the successful establishment of the cortical region, as observed in mice with arrested T cell development (48). Within each primordium, a dorso-rostralmost domain expressing *Gcm2* gives rise to a parathyroid gland from E9.5 in mice or as early as the onset of week 6 in humans, whereas a ventro-caudalmost domain identified by *Foxn1* expression produces a thymic lobe from E11.25 in mice or mid-week 6 in humans (Figure 2B) (44, 49–52). Epithelial cell proliferation fills the pharyngeal pouch lumen by forming cord-like structures with smaller lumina, similar to branching morphogenetic events in other organs (Figure 2C) (47). In this context, activation of Foxn1 blocks the respiratory development (53) and, along with subsequent colonization by lymphocyte precursors, seems to be responsible to produce a concentric medulla less densely cellular than the surrounding cortex (47). Fetal liver-derived lymphocyte progenitors colonize the embryonic thymus from E11.5 in mice and week 8 in humans (54, 55), whereas short-term apoptotic events around E12.0 disconnect the developing anlagen from the embryonic pharynx (41). The rudiments migrate downwards at different paces, gradually resolving the *Gcm2*- and *Foxn1*-restricted domains into two morphologically distinct structures enclosed by neural crest-derived mesenchyme (Figures 2C–F) (51, 56). Parathyroid primordia usually lag behind and move toward the tracheal region dorsally to the thyroid gland, whereas thymic rudiments move ventrally and more caudally into the thoracic cavity (Figures 2D,E). The thymic primordia ultimately fuse at the midline to produce a bi-lobed organ above the developing heart (Figure 2F). Unlike mice, humans exhibit superior parathyroid glands derived from the fourth pharyngeal pouch (Figure 2) (43), whereas organogenesis of the human thymus is essentially similar to mice both morphologically and molecularly (44). Each of these morphogenetic events during thymus organogenesis is controlled by a multitude of signals, including members of the TGF-β superfamily.

### Thymus specification and thymic epithelial cell differentiation

Early production of Bmp4 by the endoderm, the surrounding neural crest-derived mesenchyme, and the overlying ectoderm of the third pharyngeal arch and cleft raised the possibility that bone morphogenetic protein (BMP) signals may trigger thymus and parathyroid formation (57). However, conditional inactivation of *Bmp4* in both pharyngeal endoderm and mesenchyme using a *Foxg1–Cre* line had no effect in organ induction, but resulted in abnormal morphogenesis (see below) (58). This could be the result of a short-time window of 24 h necessary to establish the prospective thymic and parathyroid domains as observed in chicken embryos (59). Indeed, Patel et al. have observed using a Bmp4^lacZ^-reporter line that the onset of Bmp4 production occurred at E9.5 in the ventral pharynx close to the third pouch entrance, but not in the pouch endoderm or mesenchyme proper (57). Expression in these tissues was later achieved and expanded to the overlying ectoderm (57). The realization that endoderm patterning occurs before primitive gut and pharyngeal pouch formation still hampers the identification of signals responsible for thymus specification *in vivo* and other members of the TGF-β superfamily may also be at play (60). Particularly, activin A is required to induce definitive endoderm prior to the differentiation of third pharyngeal pouch endoderm *in vitro* (61). Since gene targeting of some superfamily ligands or their receptors results in embryonic lethality (62–64), new conditional mutants should be produced taking into consideration that gene deletion may have to occur earlier and at different embryonic compartments than previously thought.

The possibility that thymus induction depends on synergistic effects of TGF-β superfamily ligands with non-superfamily signals is a likely case (59). Endoderm-derived undifferentiated epithelial cells comprise a homogeneous population phenotypically defined as cytokeratin (K)5^+^K8^+^EpCAM^+^MTS24^+^ in the thymic primordium of mouse embryos at E12.0 (65). When a single progenitor cell labeled with enhanced yellow fluorescent protein (eYFP) was microinjected into an unlabeled syngeneic thymus rudiment with the same age, and transplanted under the kidney capsule, both cortical and medullary portions showed scattered eYFP^+^ TECs also positive for region-specific markers after 4 weeks, revealing that common bipotent progenitors are able to produce both epithelial lineages during embryogenesis (65). Recently, thymic epithelial progenitor cells (TEPCs) bearing stem-cell features were also identified in the thymus of adult mice as a MHCII^low^α6 integrin^high^Sca-1^high^ subset (66). They mature in a highly complex stepwise process not fully understood, ultimately producing cortical TECs (cTECs) or mTECs (67).

Cortical TECs are sparsely distributed and may be identified as CD45^−^EpCAM^+^Ly51(CD249)^+^*Ulex europaeus* lectin 1 (UEA-1)^−^K5^−^K8^+^ cells with high levels of both MHC II and the proteasome subunit β5t (68–71). Considering the TGF-β-related pathways, cells from neonatal mice express both the *Acvr2a* (ActRII) and *Acvr2b* (ActRIIB) genes for the common receptors, in addition to *Acvr1* (Alk2), *Bmpr1a* (Alk3), and *Bmpr2* (BMPRII) for the BMP-specific receptors, and the TGF-β-specific type I receptors, Alk4 (*Acvr1b*) and Alk5 (*Tgfbr1*), and type II receptor TβRII (*Tgfbr2*) (71, 72). This set of receptor genes allows cTEC to respond to both signaling branches of the TGF-β superfamily, even though the BMP receptor, *Bmpr1b* (Alk6), and the TGF-β receptor, *Acvr1c* (Alk7), are not present. Yet, expression of subunit genes *Inha* and *Inhbb* for inhibins and activins, *Bmp2* and *Bmp4*, and *Tgfb1* and *Tgfb3* makes possible the existence of an autocrine circuitry for thymic homeostasis, and indicate that these factors might influence early thymopoiesis (71, 72).

In the thymic medulla, mTECs are characterized by a CD45^−^EpCAM^+^Ly51^−^K5^+^K8^−^ phenotype with variable levels of UEA-1, MHCII, CD80, and Aire (67). These distinct expression profiles seem to take part in the differentiation program in which MHCII^high^CD80^high^ mature mTECs expressing Aire are responsible for the production of numerous peripheral self-antigens in the thymus, a critical event for central tolerance (67, 73–77). Hence, SP cells that strongly interact with self peptides through MHC molecules (pMHC) arrest migration, exhibit sustained TCR activation, persistent high levels of cytosolic Ca^2+^, and early caspase activation, leading to macrophage-dependent phagocytosis (78, 79). Surprisingly, thymocyte apoptosis triggers the production of all three TGF-β ligands by dendritic cells (DC), macrophages, and TECs in the medullary region of neonate or adult thymuses, a phenotype that was partially impaired in *Bim* mutants (80). In addition, apoptosis-driven production of TGF-β signals resulted in an increased generation of thymic regulatory T (tTreg) cells (see below) (80). Interestingly, mTECs are the cell type in the thymus that express most ligand genes of the TGF-β superfamily and their cognate receptors – *Inha* and *Inhbb*, *Bmp2*, *Bmp3*, *Bmp4*, *Bmp5*, *Bmp6*, and *Gdf6*/*Bmp13*, *Gdf3*, *Gdf6*/*Bmp13*, *Gdf8*/*myostatin*, *Gdf10*, *Gdf11*, and *Gdf15*, *Lefty1* and *Lefty2*, *Tgfb1*, *Tgfb2*, and *Tgfb3* along with *Acvr2a* (ActRII), and *Acvr2b* (ActRIIB), *Acvr1* (Alk2), *Bmpr1a* (Alk3), and *Bmpr2* (BMPRII) for BMP/growth and differentiation factor (GDF) signaling, and *Acvr1b* (Alk4) and *Tgfbr1* (Alk5) for the TGF-β/Activin/Nodal pathway, in addition to the type III receptor gene *Tdgf1* (Cripto) (71, 72, 81, 82).

The possibility that members of the TGF-β superfamily produced by mTECs may influence T cell differentiation or impact thymus physiology cannot be ruled out and remains to be thoroughly investigated. For instance, despite the previously identified BMP ligands in mTECs – Bmp3/osteogenin, Bmp5, Bmp6, and Bmp13 – there is no available functional information regarding their activities in the thymus to our knowledge. It is known, on the other hand, that Bmp6 exerts an antiproliferative effect in peripheral CD19^+^ B cells and induces apoptosis in CD27^+^ memory B cells (83). By contrast, *Tgfbr2* deficiency in differentiating T cells increased apoptosis of TCRβ^high^CD4^+^ and TCRβ^high^CD8^+^ mature SP cells after anti-CD3 treatment or of TCRβ^high^OT-II T cells after antigen-dependent stimulation, thus revealing that TGF-β signals might be involved in thymocyte-negative selection (84). Interestingly, loss of *Tgfbr2* in TECs using a *Foxn1–Cre* mouse line resulted in an expansion of the mTEC compartment – especially MHCII^high^ cells – without affecting cTEC cellularity and the morphology of the corticomedullary junction (85). Indeed, other lymphocyte-derived signals than TGF-β ligands are known to influence mTEC maturation, a phenomenon that is largely known as “thymic cross-talk” (86).

Signaling by TGF-β superfamily members appears to play a secondary role in regulating a master regulator of thymus development and function. Inactivation of the transcription factor Foxn1 results in an athymic phenotype despite the formation of an epithelial anlagen during embryogenesis (49, 87). Expression of *Foxn1* in thymic primordia is anticipated by the production of Bmp4 and Wnt4 in the epithelium and the adjacent mesenchyme of the third pharyngeal pouch from E10.5 in mice and from mid-week 6 in human embryos (23, 57, 88). Accordingly, *in vitro* treatment of fetal thymic organ culture (FTOC) with BMP4 or overexpression of *Wnt4* in a TEC cell line upregulated the expression of *Foxn1* (88, 89). However, conditional inactivation of *Bmp4* in the pharyngeal endoderm and mesenchyme did not affect *Foxn1* expression (58), similarly to transgenic embryos expressing the BMP-antagonist *Noggin* in TECs (90). In turn, information on blockage of Wnt4 and its effect over the expression of *Foxn1* is limited. In particular, Talaber et al. have shown that a single administration of dexamethasone caused the reduction of both Wnt4 and Foxn1 levels (91). Interestingly, conditional deletion of β-catenin in mTECs using a *BK5–CreER^T^* line resulted in *Foxn1* downregulation (92). Altogether, the available evidence suggests that induction or maintenance of such an essential transcription factor in the thymic epithelia relies on an intricate molecular hierarchy with a key participation for BMP and WNT signals, which may provide some kind of redundancy for TEC differentiation and function.

With a great potential for translational medicine, differentiation of TEPCs from mouse or human embryonic stem cells (ESCs) can be achieved under culture conditions by the addition of selected growth factors, including TGF-β superfamily ligands. For instance, Lai and Jin have initially reported that incubation with Fgf7, Bmp4, Egf, and Fgf10 produced K5^+^K8^+^EpCAM^+^ cells from mouse ESCs (93). These cells were able to further differentiate into medullary K5^+^K8^−^ and cortical K5^−^K8^+^ TECs when transplanted with CD4^−^CD8^−^CD45^+^ thymocytes under the kidney capsule and sustain normal T cell maturation (93). In humans, an Activin A-dependent inductive stepwise process first differentiate ESCs into definitive endoderm (94), and later into *SOX2*^+^*FOXA2*^+^*CDX2*^−^ anterior foregut endodermal cells by the concurrent inhibition of BMP and Activin/TGF-β signaling using Noggin and the type I receptor-specific inhibitor SB-431542, respectively (95). Further development into TEPC may be achieved by relatively similar approaches, generally modulating retinoic acid, canonical Wnt, and BMP level (61, 96).

### Thymus colonization by lymphoid precursors

Colonization of the thymic primordia occurs through intermittent cell flow based on chemokine-dependent mechanisms (55, 97–100). It begins discretely prior to organ vascularization with T cell-restricted progenitors that are unable to definitely populate the thymus (55, 97). Cell influx is transiently interrupted during thymus migration to the thoracic cavity (42). Then, a second wave of cell colonization brings multipotent T cell- and NK-cell progenitors before birth (55). The most significant chemokines currently identified for attracting early T lineage progenitors (ETPs) to the developing avascularized thymus are CCL25 and CCL21 (98). Curiously, whereas CCL25 is produced by both *Foxn1*-positive TECs and the adjacent parathyroid primordium, CCL21 is expressed only by *Gcm2*-positive cells (99, 101). These ligands signal, respectively, through the CCR9 and CCR7 receptors present in CD45^+^ ETPs (102–105). However, it is still poor defined whether members of the TGF-β superfamily directly or indirectly influence or are modulated by these chemokines during thymus colonization. In particular, Gordon et al. observed delayed ETP homing into *Bmp4*-deficient thymic primordia at E11.5, but no significant differences in CCL25 expression in relation to wild-type thymus (58). The relationship with CCL21, other chemokines and their cognate receptors in the embryonic thymus, if present, remains to be determined. Of note, many pathological conditions and morphogenetic events show participation of TGF-βs, BMPs/GDFs, and activins/inhibins in the modulation of chemokine production and *vice* *versa* (106–113).

Interaction of immigrating lymphocyte progenitors with the thymic stroma is critical for adult thymus organization, but not for TEC differentiation during embryonic development. Using CD3ϵ transgenic mouse embryos, known to exhibit arrested T cell maturation at the triple negative (TN) CD3^−^CD4^−^CD8^−^CD44^+^CD25^−^ ETP stage (114, 115), Jenkinson et al. have shown that K5^+^K8^+^ bipotent TEPCs normally differentiate into functional K5^+^K8^−^ medullary and K5^−^K8^+^ cortical TECs, although adult thymus in these transgenic animals exhibit persistent flat organization with morphologically abnormal cortex (115, 116). In particular, transfer of normal bone marrow cells into RAG2^−/−^; tgϵ26 chimeric mice, in which bone marrow cells from mice mutant for the recombination activating gene 2 (RAG2) were previously transplanted into newborn tgϵ26 mice, rescued thymic organization and cellularity in the adult (48).

### Thymus migration

The subsequent migration of the thymus into the thoracic cavity also relies on signaling by members of the TGF-β superfamily and depends on neural crest cells. Despite a minor contribution in thymus cellularity, forced production of the BMP-antagonist Noggin in the caudal hindbrain prior to neural crest migration using *B2-NC:Noggin* transgenic mice culminated in thymic hypoplasia or aplasia later in development (117). Indeed, Bmp2 induces Cdc42-dependent actin cytoskeleton reorganization and filopodia formation in neural crest cells, consequently affecting their subsequent migration (118). Moreover, conditional loss of *Bmp4* in mice expressing *Foxg1–Cre* impaired the separation between correctly patterned parathyroid and thymus, which also exhibited a partially compromised capsule (58). Yet, based on observations performed for thyroid migration (119), Gordon and Manley have proposed that the downward migration of the thymus may be driven by signals from the pharyngeal blood vessels, more specifically the carotid arteries (Figures 2D–F) (42). Remarkably, mouse embryos with cardiac neural crest cells deficient for the type I receptor Alk5 (*Tgfbr1*) show defective cardiac outflow development, with atypical branching of carotid arteries and failed migration of still connected parathyroid and capsule-encased thymus (120). This raises the possibility that the directional cue for thymus migration might be Alk5 ligand (e.g., TGF-β1–3 or Gdf11), possibly secreted or released through the endothelium (Figures 2D–F). By contrast, conditional inactivation of *Tgfbr2* in TECs by a *Foxn1–Cre* mouse line does not affect thymus final positioning (121). Although producing distinct phenotypes, each signaling branch by members of the TGF-β superfamily is involved in the downward migration of thymic primordia and reveals a critical, but still poorly understood role for the neural crest-derived capsule during thymus organogenesis. Neural crest cells may also differentiate into endothelial cells, pericytes, and smooth muscle cells, and were found to persist in adult mice up to the onset of thymus involution (122).

## Thymus Organization and Maturation of T Cells Under TGF-β Superfamily Signals

The adult thymus exhibits two gross anatomical regions easily identified by their histological staining patterns. The peripheral cortex harbors more immature and mostly small thymocytes, and is darker-stained due to a higher cell density. A corticomedullary junction supplied by numerous septal blood vessels makes the transition between the cortex and the central medulla. This latter region is paler due to cell size and a lower T cell density (123–125). A capsule of connective tissue encases the organ. It consists of an outer layer rich in type I collagen and an inner layer of reticular fibers containing type III collagen, and projects type I collagen-containing septa into the parenchyma, partially subdividing the thymus into smaller lobules (126).

**Figure 3:**
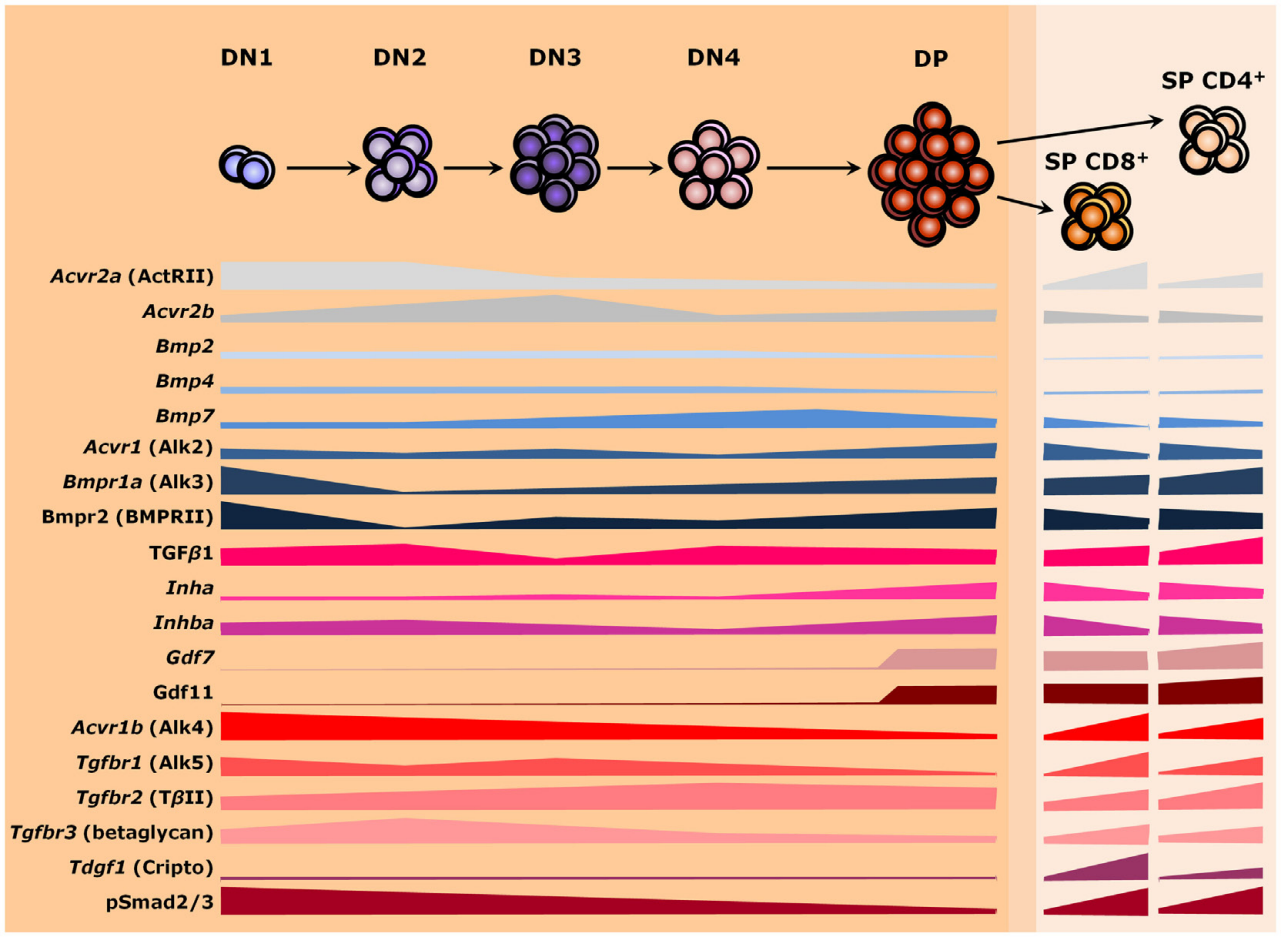
**TGF-β superfamily during thymopoiesis**. Levels of selected ligands, receptors, and Smad intracellular effectors during the differentiation of αβ T lymphocytes. Common receptors between the TGF-β and the BMP branches are colored in shades of gray, whereas components of the BMP and the TGF-β pathways are colored in shades of blue and red, respectively. The darker orange region of the scheme represents the thymic cortex, whereas light orange represents the thymic medulla. A thin corticomedullary region is represented in between the cortex and medulla. Omitted components are either not present during thymocyte maturation or no information is available at present. DN, double-negative; DP, double-positive; SP, single-positive.

Signals from members of the TGF-β superfamily have a major influence on T cell differentiation and thymus homeostasis. As secreted molecules, they may be locally produced by thymic stromal cells and act over developing T cells as paracrine factors or be produced by the thymocytes themselves and work autocrinely. Alternatively, factors from the developing T cells may similarly operate over stromal cells to support thymus homeostasis. However, thymocytes do not express most members of the TGF-β superfamily and the ones present vary in expression as cells differentiate (Figure 3). Similar changes are also found for receptor genes (127). Such differences in gene expression occur during T cell maturation, but also when comparing the same stage from fetal and adult thymuses (127–131). Nevertheless, provision of soluble growth factors seems to rely mostly to stromal cells, particularly TECs (71, 127). It is still possible that members of the TGF-β superfamily also act over large distances, being produced by other organs and reaching the thymus through the circulatory system (132). The importance of endocrine stimuli for intrathymic T cell maturation has been largely investigated (133), but whether a given TGF-β ligand exerts long-range effects over thymopoiesis remains to be properly addressed.

Changes in phosphorylation levels of Smad2/3 (pSmad2/3) and Smad1/5/8 (pSmad1/5/8), respectively, used as read-outs for the activities of TGF-β/Activin/Nodal and BMP/GDF signaling, follow differences in the expression of respective cognate receptors as thymocytes mature (134, 135). Thymocytes differentiate in a stepwise process that involves the somatic rearrangement of T cell receptor (TCR) genes while migrating in close contact with stromal cells and the extracellular matrix (ECM) throughout thymic compartments (136, 137). In this process, a major group of αβ TCR-bearing T cells are produced, which ultimately function by recognizing peptide antigens presented by class I or class II major histocompatibility complexes (MHC I or MHC II, respectively) on the surface of host cells (138). Alternatively, a distinct lineage of T cells bearing γδ TCR chains develop, which recognize a quite unique group of molecules (139). Noteworthy, intrathymic lineage restriction and cell fate are determined not only by the type of TCR and its avidity for self-antigens but also by the acquisition of co-receptors that relay signals to intracellular effectors during T cell activation. Hence, generation of distinct cell types are tightly controlled as thymocyte progresses through thymic niches (7). Herein, we will point out some key aspects of the expression and influence of TGF-β superfamily signaling molecules on the distinct paths of thymocyte development: from CD4^−^CD8^−^ DN T cell precursors (further subdivided in DN1 to DN4 stages based on the surface expression of CD44 and CD25) to the highly expanded immature CD4^+^CD8^+^ DP cells, and upon the mature CD4^+^CD8^−^ or CD4^−^CD8^+^ SP cells.

### DN1 to DN2 cells

Entry of bone marrow-derived Lin^−^cKit^high^CD44^+^CD25^−^ cells, or ETPs, into the thymus occurs through the corticomedullary junction. In this intermediate region, these immature cells with T cell–B cell–myeloid potential come into contact with K5^+^K8^+^ bipotent TEPCs and mature T cells (67, 124, 140). They subsequently move into the thymus cortex toward the subcapsular zone as DN cells, as defined by the lack of CD4 and CD8 co-receptors (7, 141). In the cortex, developing thymocytes then upregulate CD25 – the α chain of the IL-2 receptor – to become CD44^+^CD25^+^ DN2 cells, which undergo D_β_ to J_β_ recombination of the β-chain locus (142, 143). This DN1 to DN2 transition is accompanied by a strong downregulation of *Bmpr1a* (Alk3) and *Bmpr2* (BMPRII) expression (127, 129). Most cells at this stage present high levels of pSmad2 along with Alk4 (*Acvr1b*) and ActRII (*Acvr2a*) on their cell surface, although a few cells also exhibit Alk5 (*Tgfbr1*) and TβRII (*Tgfbr2*) receptors (134). DN cells also express the type III co-receptor betaglycan/TβRIII (*Tgfbr3*), with highest levels at DN3 cells (144). Betaglycan seems to increase the binding strength of some ligands with their cognate receptors, therefore potentializing their effects (145–147). Thymocytes express no *Bmpr1b* (Alk6), *Acvr1* (Alk2), and *Acvr1c* (Alk7) during thymopoiesis (127, 129). Yet, high levels of inhibin βA subunit (*Inhba*) and TGF-β1 (*Tgfb1*) contrast with reduced levels of the inhibin α subunit (*Inha*), Bmp2, Bmp4, and Bmp7 at the DN2 stage (81, 82, 127, 130). When *Inha* mutants were used for E14.0 FTOC, a partial arrest at the DN2 stage impaired further T cell maturation (148). Likewise, antibody-dependent blocking of betaglycan in E14.0 FTOC resulted in a reduction of both DN2 and DP cells (144). By contrast, addition of TGF-β1 or TGF-β2 in E14.0 FTOC strongly inhibited T cell development by mainly impairing the differentiation of DN1 cells into DN2 (149). A slightly less strong impact after BMP4 treatment of E15.0–E15.5 FTOC or suspension cultures of fetal thymocytes resulted in cell cycle arrest at the DN1 stage without induction of apoptosis (89, 150). The use of BMP4-treated chimeric human–mouse FTOC produced similar findings (81), revealing a conserved role for Bmp4 during evolution. Besides, partial redundancy between BMP ligands also seems to occur in the thymus, since treatment of FTOC with BMP2, but not with BMP7, similarly affected the production of DP cells (150).

### DN2 to DN3 cells

Following T cell differentiation into CD44^−/low^CD25^+^ DN3 cells, V_β_ to DJ_β_ recombination gives rise to the β chain of the pre-TCR (143). At this stage, the levels of *Inha*, *Bmp2*, and *Bmp4* remain relatively low, *Bmp7* becomes upregulated up to the CD3^−^CD8^+^ intermediate single-positive (ISP) stage, and expression of *Inhba* and *Tgfb1* declines (82, 127, 130). Levels of Alk4 (*Acvr1c*), Alk5 (*Tgfbr1*), and ActRII (*Acvr2a*) gradually reduce as thymocytes mature, in contrast to TβRII, which is slowly upregulated – at this stage, Alk4 and Alk5 are co-expressed (134). Expression of *Bmpr1a* (Alk3) and *Bmpr2* (BMPRII) presents a small recovery at the DN3 and DN4 stages (127, 129). Nevertheless, conditional inactivation of *Bmp7* in the hematopoietic lineage using a *vav-iCre* line had no significant impact on T cell differentiation and total cell numbers, likely because endoderm-derived cTECs and mTECs may supply enough Bmp7 or other redundant factor for the mutant thymocytes (71, 82, 150). In particular, subcapsular cTECs, cortical DCs, and mTECs express *Bmp2* and *Bmp4* (71, 81, 82). Activation of the Bmp4 pathway in stromal cells indirectly impacts the DN to DP transition, as revealed by reconstitution experiments with thymocyte-depleted stroma treated with BMP4 or untreated stroma with BMP4-treated DN cells (89). Of note, although highly expressed up to the transition from DN2 to DN3, being downregulated up to the DP stage, and sustained at low levels at SP subsets (127), the gene referred as *Bmp1* is a procollagen C-proteinase involved in ventral body wall closure during embryogenesis. To our knowledge, there is no available functional information regarding its role during thymopoiesis, except that it was also found in cTECs and mTECs (71, 151).

### DN3 to DN4 cells

Should rearrangements result in unproductive β chains, DN3 cells undergo apoptosis and are phagocytized by cortical macrophages or DCs in a process termed β-selection (143, 152). Otherwise, successful recombination leads to a reduction in CD25 expression and the expansion of CD44^−^CD25^−^ DN4 thymocytes (153, 154). Both activin A and inhibin A similarly stimulate the DN3 to DN4 transition, as revealed in FTOC from wild-type fetuses at E14.0. However, treatment with activin A led to higher numbers of mature CD24^low^CD8^+^TCRβ^high^ T cells at the expense of CD4^+^ cells, in contrast to inhibin A treatment, which stimulated the transition from DN4 to DP cells (148).

### DP cells

Rearrangement of the TCRα chain occurs at the DP stage and cells move from the cortical zone toward the thymic medulla (143, 155). During this migration, cTECs present self peptides through MHC molecules (pMHC) to the TCR of intermingling DP thymocytes in a process known as positive selection, in which interactions of low-avidity drive clones to survive and continue maturation (156). At the DP stage, Alk4 (*Acvr1b*), Alk5 (Tgfbr1), and ActRII (*Acvr2a*) reach their lowest levels, but the number of cells concomitantly presenting Alk5 and pSmad2 increases in relation to Alk4-positive cells (134). By contrast, *Bmpr1a* (Alk3) and *Bmpr2* (BMPRII) are highly expressed (127, 129). Two members of the GDF subgroup, Gdf7 and Gdf11, seem to be induced in DP cells and sustained at SP stages, with CD4^+^ T cells presenting relatively higher levels than CD8^+^ T cells (127). Gdf7 signals through BMP-specific receptors as Alk3 and BMPRII, whereas Gdf11 binds TGF-β-related receptors, as Alk4 and Alk5 (157–159). Their roles on T cell function are largely obscure, if any. Mouse mutants for *Gdf7* exhibit variable hydrocephalus and fail to produce a class of commissural neurons (160). Male mutants are sterile due to impaired differentiation and branching morphogenesis of the seminal vesicle, with no other affected reproductive structure (161). In turn, mutants for *Gdf11* show homeotic transformations due to a delayed trunk to tail transition (162, 163). They die after birth because of renal defects, which may vary from hypoplasia to complete bilateral agenesis (164). Curiously, oral infection with Gram-negative bacteria, *Aggregatibacter actinomycetemcomitans*, in rats led to a chronic upregulation of *Gdf11* expression among other cytokines in both peripheral CD45RA^+^CD4^+^ T cells and B cells (165). At present, however, little is known on the effects of GDFs over thymopoiesis.

### SP cells

Still in the cortex, differentiating thymocytes start to lose the expression of either CD4 or CD8 and migrate toward the medulla. The choice for either CD4 or CD8 SP lineage seems to occur at a transitional step defined as CD4^+^CD8^low^ and depends on TCR interaction with the MHC class II or class I, respectively (166, 167). Additionally, it also relies on the triggering of a transcriptional machinery that operates distinctly for final differentiation (165, 166). Noteworthy, the SP cells sustain *Bmpr1a* (Alk3) and *Bmpr2* (BMPRII) expression, and upregulate Alk5 (*Tgfbr1*) and TβRII (*Tgfbr2*), which lead to increased levels of pSmad2 (84, 127, 129, 134). At this stage, fine-tuning of TGF-β signaling may occur by type III co-receptors – CD4^+^CD8^−^ cells upregulate *Tgfbr3* (betaglycan), whereas CD4^−^CD8^+^ cells exhibit higher levels of Cripto (*Tdgf1*) (127, 144). Genetic loss of *Tgfbr3* in FTOC resulted in decreased numbers of both DP and SP cells, probably related to the high rates of apoptosis in DN, DP, and CD4^+^ SP subsets (144). An apoptotic phenotype was also observed in the liver of *Tgfbr3* mutants (168). However, a functional role for Cripto during thymopoiesis is currently unresolved, despite its importance for TGF-β binding and inhibition (169). Mutants for this gene present a strong deleterious phenotype during gastrulation and die shortly afterward (170, 171). Modulation of TGF-β family members, their receptors, and co-receptors at the DP stage is therefore associated with the terminal differentiation of thymocytes.

## Impact of TGF-β Signals on the Differentiation of Thymic Regulatory T Cells

Regulatory T (Treg) cells have the ability to suppress autoreactive T cells, and they can originate from the thymus or be induced in the periphery (172). Thymus-derived Treg (tTreg) arise in the thymus from SP CD4^+^ T cells that escape negative selection during maturation by presenting TCR signals of variable affinities (80, 172–174). More specifically, TCRs with high avidity for self-antigens trigger a new upregulation of CD25 (IL-2 receptor α chain) and therefore exhibit an increased responsiveness to IL-2, ultimately inducing the expression of the transcription factor forkhead box P3 (*Foxp3*) through a STAT5-dependent mechanism (175–177). Foxp3 is the critical transcription factor for Treg cell lineage, as its loss abolishes tTreg cells and lead to systemic autoimmunity and death (178, 179). Conversely, forced expression of *Foxp3* in CD25^−^CD45RB^high^CD4^+^ SP cells transferred into severe combined immunodeficiency (SCID) hosts suppressed exacerbated inflammation (180). Unlike previously thought (181), however, expression of *Foxp3* in developing tTreg cells induced apoptosis instead of cell survival. Cell death is prevented by limiting concentrations of γc-mediated survival signals enough to sustain only fewer than one million Foxp3^+^ cells (182).

Signals from members of TGF-β superfamily also play important roles over the differentiation and survival of tTreg cells. In particular, conditional loss of *Tgfbr1* (Alk5) in thymocytes seems to be involved in tTreg specification, since a *Lck–Cre* mouse line completely blocked differentiation of tTreg cells in neonatal mice, whereas later inactivation of *Tgfbr1* by a *Foxp3–Cre* line produced no differences in tTreg numbers as compared to wild-type mice (80, 183). In addition, the intrathymic injection of an anti-TGF-β antibody suppressed *Foxp3* expression in a TCR transgenic CD4^+^CD25^−^ SP cells (80). Of note, impaired Alk5 signaling induced by the *Lck–Cre* line caused no significant impact on CD4^+^ and CD8^+^ SP cell numbers (183). A later increase in Treg cells induced in the periphery (pTreg) in these mutant mice relied on IL-2 signaling, since ablation of this cytokine produced no detectable cells in organs, such as the spleen and liver (183). Similarly, thymocyte deficiency of *Tgfbr2* from a *CD4–Cre* mouse line resulted in reduced numbers of tTreg cells due to Bim-dependent apoptosis likely independent of γc-signaling, without affecting TCR-β^high^CD4^+^Foxp3^−^ mature T cells in neonatal mice (84). Unlike *Tfgbr1*-mutant thymocytes, conditional deletion of *Tgfbr2* also resulted in low numbers of pTreg cells (84). Induction of pTreg cells relies on the Smad3-dependent upregulation of *Foxp3* triggered by activation of both TCR and TGF-β signaling and facilitated by retinoic acid, which increased pSmad3 accessibility to regulatory sequences of the Foxp3 promoter and concurrently counteracted the suppressing effects of a c-Jun N-terminal Kinase (JNK) inhibitor (184, 185). Genetic analyses of the regulatory CNS1 region of *Foxp3*, which contains binding sites for NFAT, Smad3, and RAR/RXR, revealed that tTreg cell development occurs independently of its activation, whereas its chromosomal deletion largely impaired the production of pTreg cells in secondary lymphoid organs (184–186). In accordance to the different requirements revealed for tTreg in comparison to pTreg populations, TGF-β1 is essential for the peripheral differentiation and maintenance of pTreg cells, but seems to be dispensable for tTreg maturation (187).

Taking into consideration the upregulation of all three TGF-β ligands by stromal cells upon thymocyte apoptosis in the thymus, along with recent findings regarding mutants for distinct TGF-β-specific receptors (80, 84, 183), it is possible that TGF-β ligands may play a redundant yet underestimated role in the immune system. Noteworthy, mutants for TGF-β2 and TGF-β3 also exhibit perinatal mortality, a characteristic that complicates the examination of their role in adults (188–190). Although at first sight, the phenotypes observed in these mutants were generally non-overlapping, some particular structures showed similar defects between single mutants (e.g., cleft palate in either TGF-β2 and TGF-β3 mutants) or exclusive abnormalities in compound mutants, such as abnormal brain vascular morphogenesis and impaired midline fusion along with earlier embryonic lethality in *Tgfb1^RGE^;Tgfb3* and *Tgfb2;Tgfb3* compound mutants, respectively (191, 192). However, development of tTreg was never evaluated in these compound mutants. An alternative explanation may consider the participation of a previously unappreciated ligand of the TGF-β superfamily in the differentiation of tTreg cells. Whether this is indeed the case, this candidate ligand should probably signal through Alk5 and TβRII receptors to phosphorylate Smad2 and Smad3 intracellular effectors. Thereby, likely ligands to be thoroughly evaluated due to their expression pattern and receptor affinity are Gdf11 and Gdf8/myostatin – curiously two members that showed redundancy in patterning the axial skeleton as revealed by *Gdf11;Mstn* double mutants. Unfortunately, examination of fetal thymus morphology and T cell differentiation using FTOC was not performed in these mutants (193).

Noteworthy, TGF-β signals also regulate the thymic development of IL-17-producing cells. A subset of γδ T cells acquire the capacity to produce IL-17 inside the thymus via a TGF-β1-dependent machinery, and both Tgfβ1^−/−^ and Smad3^−/−^ mice were shown to be completely devoid of IL-17-producing γδ T cells (194). Additionally, NKT17 cells comprise a thymic-derived IL-17-producing, CD1d-restricted, and glycolipid antigen-reactive T cell subset (195, 196). These cells express high levels of TβRII and depend on TGF-β signals for differentiation and survival within the thymus and in the periphery (197, 198).

## A TGF-β Member for Thymus Rejuvenation?

Aging is an inherent process of living beings, normally associated with gradual loss of function and structure over time – accumulation of reactive species, DNA damage, abnormally folded proteins, and telomere shortening are just some of the molecular changes that may be followed by increased apoptosis, cell transformation, or other cellular event that will ultimately lead to death (199). Although this negative scenario was initially thought to be irreversible, numerous evidences point out that at least in part it is possible to slow down or eventually reverses some specific aging phenotypes. Taking the thymus as example, aging is easily recognizable by a sharp decrease in cellularity of both lymphoid and stromal compartments, whereas the number of thymic adipocytes inversely increases (200, 201). Ultimately, these thymic changes lead to a reduction of naïve T cells in the periphery along with an increase of memory T cells, which reflects in the organism ability to respond to both infection and tumorigenesis (202).

Multiple factors may trigger thymic involution, including the production of sex steroid hormones from puberty, increased calorie intake, or diminished levels of some growth factors and cytokines, such as fibroblast growth factor 7 (FGF7)/keratinocyte growth factor (KGF), insulin-like growth factor (IGF-1), growth hormone (GH), interleukin-7 (IL-7), and IL-22 (203). Modulation of each of them is able to rescue the aged thymic phenotype and restore the immune function at some level (204–210). However, some of these strategies may be inefficient, invasive, non-specific, or produce undesirable side effects to be used in humans (211). A quest for thymic rejuvenation therapies therefore faces daunting challenges in the clinic. Of particular interest, forced expression of *Foxn1* was shown to effectively reprogram fibroblasts into TECs or regenerate fully involuted thymuses at many different experimental setups, both *in vitro* or *in vivo* (212–214). In this context, signals that control *Foxn1* expression might be used to restore the integrity of the thymic epithelial niche and subsequently flourish thymopoisesis in the elderly. In this scenario, administration of soluble factors, such as ligands of the TGF-β superfamily, may be used as regenerative drugs.

Recent findings have revealed that levels of some circulating factors vary with age and that heterochronic parabiosis, i.e., a surgical procedure that connects the circulatory systems of animals with different ages, was able to reverse age-related phenotypes as cardiac hypertrophy (132). These authors further identified the TGF-β member Gdf11 as responsible for restoring cardiac function in old mice, a finding that was further expanded to other systems. In particular, daily treatment of old mice with recombinant GDF11 improved skeletal muscle mass and strength, as well as the integrity of brain vasculature and cognitive function (215, 216). In culture, Gdf11 promoted osteoblastogenesis while inhibiting adipogenesis in bone marrow-derived cells (217). Administration of GDF11 in endothelial progenitor cells triggered cell sprouting and migration, also revealing a role in the formation of blood vessels (218).

Whether Gdf11 or other circulating factor can be used as a rejuvenating cytokine for the thymus remains to be thoroughly assessed. Indeed, *Gdf11* is expressed in the thymus of young mice (132), whereas the levels of its non-exclusive receptors, Alk4 and Alk5, vary in thymocytes and TECs, as previously discussed. Of note, however, therapy with Gdf11 produced some side effects in mice (219), and a recent study by Egerman et al. has recently questioned the aforementioned observations (220). Whereas these controversial data on Gdf11 await further investigation, it is noteworthy that heterochronic parabiosis did not reverse thymic involution, but caused atrophy with mild effects on T cell subpopulations of young mice and a reduction in the number of CD4^+^CD25^+^Foxp3^+^ regulatory T cells in old partners to the level of the young pair (221). Although a putative rejuvenating factor for the thymus still awaits to be determined, this controversial matter helps to bring the debate on the role of TGF-β superfamily members for the thymus function.

## Concluding Remarks

Although the differentiation of T cells is mainly driven by the rearrangement of TCR genes, many members of the TGF-β superfamily exert critical roles in their stepwise progression during thymic migration. Historically, special attention had been given to the activity of TGF-β ligands in the induction of Treg cells and tolerance to self-antigens, as well as to BMP signaling on thymus organogenesis. However, other members are also produced by developing thymocytes, thymic stromal cells, or may circulate throughout the body by the blood stream and reach the thymus. These ligands signal through the same limited sets of type I and type II receptors to produce dissimilar outcomes either by affecting distinct stages or cell types (e.g., thymocytes versus TECs). How such TGF-β superfamily ligands affect T cell maturation, thymus proper physiology, or its involution remain poorly understood and should be the focus of future research. In addition, a scenario in which a TGF-β superfamily member or its inhibitor acts to rejuvenate the aged thymus may be a likely case for future research.

## Conflict of Interest Statement

The authors declare that the research was conducted in the absence of any commercial or financial relationships that could be construed as a potential conflict of interest.
